# EHMTI-0209. Headache management is more than just managing pain

**DOI:** 10.1186/1129-2377-15-S1-C28

**Published:** 2014-09-18

**Authors:** T Zimmer, CB Møller, ML Westergaard, D Kjeldgaard Nielsen

**Affiliations:** 1 Department of Neurology, Danish Headache Center University of Copenhagen, Glostrup, Denmark

## Introduction

Documentation of the effect of multidisciplinary treatment in headache is needed. The newly developed DOLOtest for pain patients measuring Health-Related Quality of Life may be a valuable instrument for assessing treatment effectiveness.

## Aim

To describe the patient population referred to psychological assessment in a tertiary headache center in terms of headache characteristics and baseline DOLOtest.

## Method

All patients from the Danish Headache Center referred to psychological assessment in 2012 participated in a semi-structured interview and were given the DOLOtest. Patients were asked to score the eight domains of the DOLOtest (1 to 100 on a visual analogue scale): pain, problems with light physical activities, problems with more strenuous physical activities, problems doing your job, reduced energy and strength, low spirit, reduced social life, and sleeping problems. Higher scores indicated greater adverse impact on quality of life. Summary scores were analyzed in relation to sex and headache frequency.

## Results

215 patients were referred to psychological assessment (173 females, average age 37 years, SD 12 years). Their average headache frequency was 22 days/month (SD 10). The DOLOtest was completed by 185 patients. All eight domains were affected (range 27-59), highest mean scores were on reduced energy and strength (59), and pain (55). Patients with ≥15 days of headache/month had significantly higher scores on seven domains. There were no sex differences in all domain scores.

## Conclusion

Living with severe headache, especially if chronic, influences all aspects of patients’ perceived quality of life as measured by the DOLOtest.

No conflict of interest.

